# Proceedings of the National Medical Convention, *Held in the City of Philadelphia, in May*, 1847

**Published:** 1847-06

**Authors:** 


### RECORD OF MEDICAL SCIENCE.

### PROCEEDINGS OF THE NATIONAL MEDICAL CONVENTION,

*Held in the City of Philadelphia, in May*, 1847.

PHILADELPHIA, *May 5th*, 1847.

The delegates to the National Medical Convention assembled in the Hall of the "Academy of Natural Sciences," at 10 o'clock, A.M.

Dr. Isaac Hays, Chairman of the Committee of Arrangements from the Philadelphia delegation, opened the proceedings with a few remarks, welcoming the delegates to the city, and expressing on behalf of his delegation the gratification it afforded them to receive the members of the Convention as their guests. He then proposed a temporary organization, and nominated Dr. Jonathan Knight, of Conn., as Chairman. This nomination was unanimously confirmed.

Drs. Arnold, of Georgia, and Stillé, of Pennsylvania, were then appointed Secretaries.

On motion of Dr. Hays, and by appointment of the Chairman, Drs. Arnold, of Ga.; Blatchford of N. Y.; Thompson, of Del ; Haxall, of Va.; and Bishop, of Conn., were constituted a committee to examine and verify the credentials of the Delegates to the Convention. The meeting then took a recess for half an hour.

On re-assembling, it was moved by Dr. J. M. Smith, of N. Y., and seconded by Dr. F. C. Stewart, of N. Y., that a committee of one, from each State represented, should be appointed to nominate permanent officers for the Convention.

The following gentlemen (whose credentials had already been verified) were appointed by the Chairman:—

| | | | | | | | |
|---|---|---|---|---|---|---|---|
| 1. | Dr. | Holmes, | *Mass.* | 13. | Dr. | Moultrie, | *S. C.* |
| 2. | " | Twitchell, | *N. H.* | 14. | " | Mitchell, | *Ky.* |
| 3. | " | Dunn, | *R. I.* | 15. | " | Garvin, | *Ga.* |
| 4. | " | E. Ives, | *Conn.* | 16. | " | Buchanan, | *Tenn.* |
| 5. | " | Stearns, | *N. Y.* | 17. | " | Pierce, | *Mich.* |
| 6. | " | Hall, | *Vt.* | 18. | " | Frye, | *Ill.* |
| 7. | " | Cole, | *N. J.* | 19. | " | Carpenter, | *La.* |
| 8. | " | Norris, | *Pa.* | 20. | " | Keirn, | *Miss.* |
| 9. | " | G. W. Baker, | *Del.* | 21. | " | Bullitt, | *Mo.* |
| 10. | " | Gibson, | *Md.* | 22. | " | Shipman, | *Ind.* |
| 11. | " | Welford, | *Va.* | 23. | " | Butterfield, | *Ohio.* |
| 12. | " | Lindsley, | *D. C.* | | | | |

On motion, the Committee were instructed to nominate *four* Vice Presidents, and *three* Secretaries.

The Committee for the Verification of Credentials then presented the following report, which, on motion, was accepted, and the committee continued.

The Committee beg leave to report that they have examined the Credentials of the delegates from the several sections of the Union, and herewith submit a list of them.

Owing to the number of vouchers required to be examined, and the necessarily hurried manner in which the committee have been obliged to perform their duty, some omissions may have been made. If so, they request that gentlemen, so omitted, may at once hand in their names to the Secretaries.

R. D. Arnold,
T. W. Blatchford,
Jas. W. Thomson,
R. W. Haxall,
E. H. Bishop.

DELEGATES.

[To save space we are compelled to omit the names of the delegates.—*Ed.*]

*New Hampshire*—New Hampshire Medical Society, 5. Dartmouth Medical College, 1.

*Vermont*—Vermont Medical College, 1. Vermont Medical Society, 4. Faculty of Castleton Medical College, 1.

*Massachusetts*—Massachusetts Medical Society, 8. Middlesex District Medical Society, 3. Faculty of Medicine of Harvard University, 1. Berkshire Medical Institute, 1.

*Rhode Island*—R. I. Medical Society, 4.

*Connecticut*—Medical Institution of Yale College, 2. Connecticut Medical Society, 8. New Haven Medical Association, 1.

*New York*—College of Physicians and Surgeons in the city of New York, 3. New York State Medical Society, 19. Medical Faculty University of the City of New York, 4. Rensselaer Co. Medical Society, 2. Troy Medical Association, 1. Erie Co. Medical Association, 1. Cortland Medical Society, 1. New York Medical and Surgical Society, 5. Medical Department of the University of Buffalo, 1. Med. Fac. of Geneva College, 1. Albany Medical College, 4. New York Pathological Society, 4. New York Hospital, 2 Medical Society of the City and County of New York, 14. New York Academy of Medicine, 15.

*New Jersey*—Medical Society of New Jersey, 10. District Medical Society of Burlington, 3.

*Pennsylvania*—College of Physicians of Philadelphia, 16. University of Pennsylvania, 3. Philadelphia Medical Society, 12. Jefferson Medical College, 3. Pennsylvania College, 3. Franklin Medical College, 3. Philadelphia College of Medicine, 2. Medical Institute of Philadelphia, 1. Northern Medical Association of Philadelphia, 12. Lancaster City and County Medical Society, 7. Med. Soc. of Montgomery Co., 3.

*Delaware*—Medical Society of the State of Delaware, 9. Med. Association of the City of Wilmington, 4.

*Maryland*—Medical and Chirurgical Faculty of Maryland, 6. Association of Med. Faculty of Frederick City, 1. Washington University of Baltimore, 2.

*District of Columbia*—Medical Society of the District of Columbia, 3. Medical Department of the Columbian College, 3.

*Virginia*—Medical Convention of Virginia, 9. Medical Society of Virginia, 16. Petersburgh Medical Faculty, 5. Medical Soc. of Fredericksburg, 1.

*South Carolina*—Medical Society of South Carolina, 3. Medical College of the State of South Carolina, 1.

*Georgia*—Georgia Medical Society, 2. Medical College of Georgia, 2.

*Mississippi*—Mississippi State Medical Society, 3.

*Louisiana*—Medical Department of University of Louisiana, 3. *Missouri*—Medical Department of the St. Louis University, 2. St. Louis Association of Physicians, 1.

*Illinois*—Rush Medical College, 1.

*Indiana*—Indiana Medical College, 4.

*Michigan*—State Medical Society, 1.

*Ohio*—Medico-Chirurgical Society of Cincinnati, 1. Ohio Medical Convention, 3. Willoughby Medical College, 1.

*Kentucky*—Medical Department of Transylvania University, 3.

*Tennessee*—Medical Department of the State of Tennessee, 1.

The Committee charged with the nomination of permanent officers for the Convention, reported through their chairman, Dr. Eli Ives, the following nominations:

*President.*—Dr. Jonathan Knight, Conn.

*Vice Presidents.*—Dr. Alexander H. Stevens, N. Y.; Dr. George B. Wood, Pa.; Dr. A. H. Buchanan, Tenn.; Dr. John Harrison, La.

*Secretaries.*—Dr. Richard D. Arnold, Ga.; Dr. Alfred Stillé, Pa.; Dr. F. Campbell Stewart, N. Y.

Which were unanimously confirmed.

The officers having taken their places, the Convention was declared to be duly organized, and ready for the transaction of business.

On motion of Dr. J. L. Atlee, of Lancaster, Pa., it was—

*Resolved,* That any medical gentlemen present from States not otherwise represented, and the members of the Medical Staff of the Army and Navy, now in the city, be invited to take seats in the Convention.

The following Resolution was introduced by Dr. Thomson, of Del.

*Resolved,* That any member of the last Convention, now present, not elected a Representative in this Convention, be invited to take a seat in the same, and participate in its deliberations;—which, on being put to the vote, was lost.

The following Resolution, presented by Dr. Corbin, of Va., was also lost: viz.—

*Resolved,* Unanimously by the National Medical Convention, that the Ex-Professors of medicine of the various Medical Schools of the U. S., and such Professors from abroad as may be in Philadelphia, be invited to take seats in this body.

Dr. Hays called for the reading of the Report of the Committee appointed by the last National Medical Convention, to prepare a plan of organization for a *National Medical Association.* Dr. Watson, Chairman of the Committee, presented a report, which, after being read, was, on motion of Dr. Watson, received and laid upon the table in order to be printed.

Dr. Griscom, of N. Y., Chairman of the Committee appointed by the last National Convention to consider the expediency and mode of urging upon the State governments the adoption of measures for a registration of Births, Marriages, and Deaths presented a Report, which was received, and laid upon the table in order to be printed.

After which, on motion, the Convention adjourned to meet at 7 o'clock, P. M.

### *Evening Session.*

The Convention met pursuant to adjournment.

Invitations to the Convention to visit the Philadelphia House of Refuge and the Institution for the Deaf and Dumb, were read.

Dr. Haxall, Chairman of the Committee "on the requirements for the degree of M. D.," appointed by the Convention of 1846, presented a Report, which was, on motion, received, and laid upon the table to be printed.

The Committee on Credentials, reported the following additional delegates. From Medical Society of King's County, New York, 5. New York City Vaccine Institution, 2. The Medical Department of the University of the State of Missouri, 1. Massachusetts Medical Society, 1. Faculty of Physic of the University of Maryland, 3.

On motion of Dr. Reese, of N. Y., the verification of the credentials of the delegate from the N. Y. Vaccine Institution was referred to the delegates from that city, with instructions to report thereupon.

Dr. Couper, of Del., as Chairman, presented the Report of the Committee "on Preliminary Education," appointed by the Convention of 1846, which, after being read, was, on motion, received, and laid on the table to be printed.

Dr. Hays presented a portion of the Report of the Committee appointed by the Convention of 1846, to prepare a code of Medical Ethics, which (after some remarks by the Reporter in regard to the sources from which the provisions of the code were mainly derived) was read and disposed of temporarily, in the same manner as the foregoing reports.

The following Preamble and Resolution, introduced by Dr. Watts, of N. Y., was adopted :

*Whereas*, The credentials contain the names of delegates elected, of whom a part will not be present, therefore,

*Resolved*, That the "Register" of the Philadelphia Committee of Arrangement be adopted as the Register and Roll of the Convention.

Dr. Griscom moved that the Committee "on a Nomenclature of Diseases," of which he is Chairman, be authorized to print so much of the same, as it shall deem expedient for the use of the Convention. Carried.

Dr. D. Judkins, of Cincinnati, moved that when the Convention adjourns, it adjourn to meet at 9 o'clock, A. M. Several amendments fixing the hour at 10, and the length of the Session from 9 until 3 o'clock, were rejected, and then the original motion prevailed.

At the suggestion of Dr. Hays, it was—

*Resolved*, That no persons should have access to the floor of the Convention, except delegates furnished with the cards provided by the Philadelphia Committee of Arrangement.

After which the Convention adjourned to 9 A. M. of the following day.

*May 6th. Morning Session.*

The Convention reassembled at nine o'clock, A. M.

The Minutes of the first day's proceedings having been read and approved, the roll was called ; after which the Committee on Credentials reported the names of the following additional delegates.

From the Centre County Medical Society (of Pennsylvania,) 1. New York Pathological Society, 1. New York City and County Medical Society, 1. From the District Medical Society of Salem county, N. J., 1. From the Alumni of Castleton Medical College, Vermont, 1.

Dr. J. H. Griscom, of New York, presented the following resolution, which was adopted :

*Resolved*, That a committee of five be appointed by the Chair, to consider and report to the Convention measures for defraying its expenses.

The President then appointed the following gentlemen to the Committee : Drs. J. Bell, of Philadelphia ; A. March, of Albany ; J. M. Smith, of New York ; L. A. Smith, of New Jersey ; B. R. Wellford, of Virginia.

Dr. Stewart reported that the delegates from the New York Vaccine

Institution, whose credentials had been referred to the New York City delegations, had withdrawn the same.

Dr. N. S. Davis, of Binghamton, New York, offered the following resolution, which was ordered to be laid upon the table.

*Resolved*, That a committee of one from each State represented in this Convention be appointed by the President, whose duty it shall be to investigate the *Indigenous Medical Botany* of our country; paying particular attention to such plants as are now, or may be hereafter during the term of their service, found to possess valuable medicinal properties, and are not already accurately described in the standard works of our country; and report the same in writing, giving not only the Botanical and Medical description of each, but also the localities where they may be found, to the next annual meeting of the American Medical Association.

A letter, addressed to the Secretary of the Convention, was now received and read; it was signed by Dr. Lewis W. Chamberlayne, in behalf of the Medical Faculty of Hampden Sydney College, at Richmond, Va., and expressed the concurrence of the Faculty in the objects that brought the Convention together, and a regret that the delegate appointed to represent that body, was prevented by illness from attending.

An invitation was received from the Rev. Jno. A. Vaughan, Principal of the "Pennsylvania Institution for the Instruction of the Blind," for the members of the Convention to visit that Institution.

On motion of Dr. R. S. Kissam of N. Y., this, and all other invitations extended to the Convention, were ordered to be acknowledged and accepted by the Secretaries.

Dr. J. Bell, Chairman of the Committee appointed by the Convention of 1846, to prepare a code of Medical Ethics, read the introduction to the report presented yesterday, which was, on motion, ordered to be printed.

The report of the Committee appointed by the last Convention, to consider Dr. Bartles' Resolution, in regard to the union of teaching and licensing, was now called for. Dr. McNaughton, of Albany, Chairman of the Committee, presented and read a minority report. The majority report was presented by Dr. Isaac Parrish of Philadelphia.

On motion of Dr. Reese, of N. Y., both reports were received and ordered to be printed.

Dr. A. B. Shipman, of N. Y., presented the following which was adopted:

*Resolved*, That Dr. Thomas Spencer, of Geneva, N. Y., accidentally with us on a tour for the restoration of his health, be invited to sit with us as a member of this Association; to participate in the debates but not to vote.

Dr. J. H. Griscom, of N. Y., Chairman of the Committee appointed to prepare a "Nomenclature of Diseases," presented and read the report of his committee; which was received and ordered to be printed.

The following order was proposed by Dr. J. V. C. Smith, of

Boston, and, on motion of Dr. Watson, laid upon the table for the present.

*Ordered*, That a committee be appointed by the chair to select a place for the National Medical Convention to hold a session in 1848.

Dr. Couper, of Delaware, moved that the report of the Committee on Preliminary Education, be now taken up. This motion prevailed, and the report having been again read, the resolutions accompanying it, were declared to be under consideration.

Several amendments to the first resolution having been proposed and lost, the question was taken upon the adoption of the resolution: and it was adopted.

The second Resolution was adopted after substituting the word "pursue" in place of "complete."

The third Resolution having been read, Dr. Cabell, of Va., offered an amendment, which was adopted, but subsequently reconsidered and withdrawn, and the resolution passed without amendment.

On motion, the Report of the Committee was adopted and ordered to be printed, together with the resolutions, as part of the proceedings of the Convention.

On motion of Dr. John Stearns, of New York, Professor Hare, of Philadelphia, was invited to a seat in the Convention, and to take part in the debates, but without the privilege of voting.

An invitation was received from the delegates in attendance from the city of Baltimore, for the National Association about to be formed, to hold its Session for 1848 in that city.

On motion, it was determined that when the Convention adjourns, it will adjourn to meet again at 5 o'clock, P. M.

Dr. Joel Hopkins, of Maryland, offered the following Resolution to be adopted as a rule of the Convention.

*Resolved*, That no gentlemen shall be permitted to speak more than twice on the same proposition, nor occupy more than fifteen minutes in speaking, without leave of the Convention.

The resolution was amended, by inserting "ten minutes" in lieu of "fifteen minutes," and then adopted.

On motion of Dr. M'Naughton, of Albany, 250 additional copies of the Report of the Committee on Preliminary Education were ordered to be printed, and the Convention adjourned.

*Afternoon Session.*

The Convention was called to order at the hour to which it had adjourned.

The Committee on Credentials reported the name of Dr. J. Breitenbach, as an accredited Delegate from the Lebanon County Medical Society of Pennsylvania.

Dr. J. L. Pierce, of Michigan, offered the following resolution, which was adopted.

*Resolved*, That the delegates to this Convention, be requested to ascertain as far as may be practicable, and report to the next annual meeting, the number of practitioners of medicine in their respective states—designating the number who may have received a diploma from a medical college, the number who may have been licensed by a medical society, and the number who practice medicine without any authority whatever.

Dr. I. Hays, of Philadelphia, moved to take up the report of the committee, appointed under the fourth resolution of the convention of 1846. This motion prevailed, and on motion of Dr. N. S. Davis, of Binghamton, the resolutions attached to the report were ordered to be considered seriatim.

The first resolution having been read, Dr. Smith, of Newark, N. J., moved to amend by inserting "five months," in place of "six months"—the amendment was lost, and the question being taken upon the resolution, it was adopted.

The second, third, fourth, fifth, and sixth resolutions were then severally read and adopted without discussion.

The seventh resolution being under consideration, Dr. Washington L. Atlee, of Pennsylvania, offered the following as a substitute:

"That it is incumbent upon the Preceptors, to avail themselves of every opportunity to impart clinical instruction to their pupils; and upon Medical Colleges, to require candidates for graduation to show that they have attended upon Hospital practice for one session, whenever it can be accomplished."

This substitute, having been accepted by the committee, was adopted.

Resolutions were proposed by Drs. J. P. Jervey, of South Carolina, and Dr. A. H. Stevens, of New York, but were withdrawn in favour of the following additional resolution, offered by Dr. F. Campbell Stewart, of New York, and unanimously adopted,

"That it be suggested to the Faculties of the various Medical Institutions of the country, to adopt some efficient means for ascertaining that their students are actually in attendance upon their lectures."

The two remaining resolutions were then acted upon and passed; and on motion, the report and resolutions were adopted.

An invitation was received from Dr. Thomas S. Kirkbride, of the "Pennsylvania Hospital for the Insane," for the Delegates to visit the Institution under his control; and then the Convention adjourned to nine o'clock, A. M., on the following day.

*May 7th. Morning Session.*

The Convention re-assembled at nine o'clock, A. M.

Dr. Stevens, of New York, moved that a committee be appointed to invite Dr. John Redman Coxe to take a seat upon the floor of the Convention; which was carried.

Dr. Stearns and Ely Ives were appointed.

Dr. Blatchford, of New York, moved that Dr. Cheney Howe, of St. Louis, Mo., be invited to take a seat upon the floor of the Convention; which was carried.

The minutes of yesterday's meeting were then read.

Dr. Haxall, of Virginia, moved to reconsider so much of the minutes as relate to the fifth resolution accompanying the report of the committee of which he was chairman; which motion was carried.

He then moved to strike out the words "that not less than one hundred lectures be delivered by each Professor;" which was carried.

The resolution as amended was then adopted.

The minutes were then confirmed.

The committee on credentials reported the following additional accredited Delegates:

*Pennsylvania.*—Pennsylvania Centre Co. Medical Society, 3.

*Maryland.*—Lebanon Med. Co. Society, 12. Med. and Chirurg. Faculty of Maryland, 3.

Dr. Geo. B. Kerfoot, of Pennsylvania, offered the following:

*Resolved*, That when physicians are called upon by courts of justice to give medical evidence, or opinions in medico-legal questions; by coroners to make *post-mortem* examinations, or chemical analyses; such services shall be considered professional, and remuneration expected accordingly.

Which was laid on the table.

Dr. Sumner, of Connecticut, offered the following:

*Resolved*, That a committee of three be appointed to ascertain and report to the next meeting of this Association, what legal enactments exist in the several states relative to the practice of medicine; and also what societies and associations have been formed by the physicians of the different states, to advance the medical profession.

Which was laid on the table.

Dr. Bell, of Penn., made the following report.

The committee appointed for taking measures to defray the expenses of the Convention, Report,

That from inquiries made, they believe that the sum of six hundred dollars will cover all the expenses incident to the meeting of the present Convention, and they respectfully recommend that each member be invited to contribute three dollars to make up the above sum.

Signed, JOHN BELL, Chairman.

After some discussion the report was adopted.

Dr. L. P. Bush, of Delaware, offered the following, which was laid on the table.

*Resolved*, That the committee on expenses be requested to collect the amount indicated as necessary to defray the expenses of the Convention.

On motion of Dr. Watson, of New York, the Convention took up the report of the committee on the organization of the National Medical Association.

Dr. Naudain, of Penn., moved that it be read from the Secretary's desk.

Dr. Cock, of New York, moved to dispense with the reading.

Dr. Naudain's motion prevailed.

The report was then read by the Secretary.

Dr. Hays, of Penn. moved to adopt the resolution on page second of the printed report, as far as the words "and that" on the 12th line, which was carried.

The Convention then proceeded to the consideration, seriatim, of the articles proposed for the government of the Association.

The first article being under consideration, Dr. Kerfoot, of Penn. offered the following as an amendment, that the title be "The Conventional Association of the United States."

Which was lost.

Dr. Stout, of New York, proposed the following amendment, that the title be "The Medical Association of the United States of North America."

Which was lost.

The first article was then put to vote and adopted without amendment.

The second article being then before the Convention, Dr. Hays, of Penn., offered the following:

*Resolved*, That the report be referred back to the committee with instructions to report a plan of organization in accordance with the following sketch.

1st. The Society to consist of members to be elected by the Association, directly, or through its council.

2. Members before admission into the Association, to sign a promise to conform to the laws of the Association.

3. Members who violate this pledge, to be liable to expulsion, and to be deprived of the rights of brotherhood.

4th. For the appointment of a council to consist of the officers of the Society, and of councillors, to be elected annually, or all the former, and a portion at least of the latter, to be elected annually. The councillors to have the general superintendence of the concerns and publications of the Association, and to report the proceedings to the Association at its annual meeting.

After considerable discussion, Dr. Hays' resolution was lost.

It was then moved to adopt the second article.

Dr. Cabell, of Virginia, moved to insert after the word "Association," on the ninth line of the third page, second Article, of the printed copy, the words "but without the right of voting."

Dr. Askew, of Del., offered the following amendment, which was lost.

That the third section of the second article be amended, by adding the words "State and," between the words "each" and "local;" and that the word "ten" be stricken out, and the word "five" be inserted.

Dr. Sumner, of Conn., moved to adopt the whole of the remaining articles.

The question was then taken upon adopting the whole of the remaining articles, and was carried.

The question was then put upon the adoption in full of the report as amended, and was carried.

The Convention then took up the report of the committee on Medical Ethics.

Dr. L. P. Bush, of Del., moved to adopt the whole of the report, including the introduction.

Dr. John L. Atlee, of Penn., offered the following amendment, which was lost, viz.: in the fourth Section of Article four, to strike out the words, "in which they have been called," and to insert the words "of seniority, commencing with the youngest."

The question on Dr. Bush's motion, was then put to the Convention, and was carried.

The report of the Committee upon Registration was then taken up, and with the resolutions accompanying it, was adopted without dissent.

The Convention took a recess for ten minutes, to enable members to hand in their contributions to the committee appointed to receive them, for the purpose of defraying the expenses of the Convention. After the Convention had re-assembled, the President announced the following Committee under the 2d Resolution, accompanying the report of the Committee on Registration.

Drs. J. H. Griscom, of New York,
C. A. Lee, " "
A. Clark, " "
G. Emerson, of Pennsylvania,
R. D. Arnold, of Georgia,
Jno. D. Russ, of New York,
Lemuel Shattuck, of Massachusetts.

The following communication, from Dr. Robley Dunglison, on the publications of the Sydenham Society, was then read to the Convention.

*To the President of the National Medical Convention.*

*Philadelphia, May 6th*, 1847.

Sir,—I hope I may be pardoned by the Convention, for drawing the attention of its members to the *Sydenham Society of London*, which was instituted a few years ago, "for the purpose of meeting certain acknowledged deficiencies in existing means for diffusing medical literature," "not likely to be supplied by the efforts of individuals" It has been, and is, the purpose of the Society to carry its objects into effect by a succession of publications, embracing among others—*First*. Reprints of standard English works, which are rare or expensive. *Secondly*. Miscellaneous selections from the ancient, and from the earlier modern authors, reprinted or translated. *Thirdly*. Digests of the works of old and voluminous authors, British and foreign, with occasional biographical and bibliographical notices. *Fourthly*. Translations of the Greek and Latin medical authors, and of works in the Arabic, and other Eastern tongues, accompanied, when it is thought desirable, by the original text. *Fifthly*. Translations of recent foreign works of merit. *Sixthly*. Original works of merit, which might prove valuable as books of reference, but which would not otherwise be published, from the slender chance of their meeting with a remunerating sale;—such as bibliographies, alphabetical, and digested indexes to voluminous periodical publications, &c.

The Society was instituted in the year 1843, on the plan of the Camden, Parker, and other societies, and now consists of between two and three thousand members;—the annual subscription being in England, £1, 1s, paid in advance—in this country *five dollars*, for which sum each member receives a copy of every book printed by the Society,—the duty and expenses of transmission being, of course, paid by the member, in addition.

The edition of the first year's books, (for 1843–4,) comprising *Hecker's Epidemics, Louis on Phthisis*, and *Sydenham's works* in Latin, consisted of 1750 copies; but owing to the large increase of members, it was found necessary to reprint them, and they are now in the hands of several members in this country. Of each of the works issued for the second year—1844–5—*Paulus of Ægina*, Vol. I., *Observations on Aneurism*, and *Simon's Animal Chemistry*, Vol. I., 2250 copies were printed; yet so great was the afflux of members after the time when it was necessary for the council of the Society to determine how many copies should be printed—the number of members being then eighteen hundred—that the whole edition was soon exhausted, and for some time the council have been unable to supply the numerous applicants for the second year's books. They may, however, be reprinted, although this is uncertain.

Constituted as the officers and council of the Sydenham Society are, of many of the most distinguished members of the profession in Great Britain, whose sole object in this undertaking is the benefit of that profession, and on whom great reliance may be placed that the good work will be faithfully and ably executed, I have been emboldened to lay before the Convention its claims to their attention, and to place before them for their inspection, a copy of each of the works issued for the year 1846–7, (one other, "the Works of Harvey," being in course of distribution, but not having as yet reached this country,)—and likewise a few copies of the *Report of the Fourth General Meeting of the Society*, held on the first of May, 1846, which contains further information in regard to the proceedings of the Society, its laws, &c.

I have the honour to be, with the greatest respect,
Your obedient humble servant,
ROBLEY DUNGLISON, *Hon. Local Secretary.*

N. B.—The other honorary local Secretaries of the Society, in this country, are Dr. T. R. Beck, of Albany, and Dr. C. A. Lee, of New York, delegates to the Convention.

Dr. Stevens, of New York, moved that the report of the Committee on the Nomenclature of Diseases, which was next in order, be referred to the American Medical Association, which was carried.

The Report of the Committee on the Separation of Teaching and Licensing, was then called up.

Dr. Reese, of New York, offered the following—

*Resolved*, That the report of the majority of the committee on the subject of separating the teaching from the licensing power in Medical Colleges, be adopted by this Convention, and published in its transactions.

*Resolved*, That the report of the minority be laid upon the table, and printed in like manner.

Dr. Leonard, of Maryland, offered the following, as a substitute for the foregoing, which was accepted by Dr. Reese.

*Resolved*, That in view of the necessity hereby declared for reform in the manner of conferring degrees, the two reports submitted by the committee on the separation of teaching and licensing, be published and referred to the committee on medical education, with instructions to report at the next annual meeting of the American Medical Association, which was adopted.

The Convention then adjourned to 5 o'clock, P. M.

*Evening Session.*

At 5, P. M., the Convention met pursuant to adjournment.

Dr. J. V. C. Smith, of Boston, introduced the following Resolution, which was read and adopted.

*Resolved*, That the thanks of this Convention are due to the officers and directors of the various institutions, who have politely invited the members to visit them at their own convenience,—to the Committee of Reception and Arrangements, on behalf of the Philadelphia Delegation, for the spacious and elegant accommodations provided,—to the Academy of Natural Sciences, for the use of its Hall,—and to the whole medical profession of the city, for the marked kindness, personal attention, and generous hospitality, which have characterized their intercourse with this body since the commencement of its deliberations.

On motion of Dr. L. A. Smith, of New Jersey, it was—

*Resolved*, That those members of the Convention who left the city before the assessment of three dollars to defray expenses was made, be requested to forward that amount to Dr. J. Rodman Paul, of Philadelphia, by letter, or otherwise, as may be convenient.

Dr. Reese, of New York, introduced the following Resolution,—

*Resolved*, That a Committee be appointed to draft a memorial to the Congress of the United States, asking that a portion of the Smithsonian funds may be annually appropriated to the uses of the American Medical Association;—which was seconded by Dr. Meigs, of Philadelphia, but laid upon the table.

A like disposition was made of the following, offered by Dr. M. H. Houston:

*Resolved*, That a Committee of seven be appointed to report to the next annual meeting of the American Medical Association, such modifications of, and amendments to, the adopted plan of organization, as in their estimation may be imperiously required, in order to accomplish the objects of its original organization.

On motion of Dr. Thomson, of Delaware, it was ordered "that the names of the delegates, when published, should be arranged by States."

The following Preamble and Resolutions, introduced by Dr. Henry Carpenter, of Lancaster, were laid upon the table:

*Whereas*, The difficulties which sometimes unfortunately arise between physicians in their attendance upon the sick, are frequently owing to improper procedure, or representations on the part of the patients or friends, from an ignorance of the etiquette which should govern the conduct of the respective parties towards each other; therefore,

*Resolved*, That the President of this Convention appoint a Committee of three, to select such parts of the code of Ethics, adopted by this body, as they may deem expedient, and report the same to the Convention for approval at its Session to-morrow morning.

2d, That the Committee of Publication have a sufficient number of copies of the same printed, and deliver, or send, to each delegate a suitable number.

3d, That delegates request the editors of the public journals, in their respective localities, to publish the same as proper and useful information for the people.

Dr. Garvin, of Georgia, introduced the following Resolution, which was adopted.

*Resolved,* That the thanks of the Convention be presented to their President, Dr. Knight; to the Vice Presidents, Drs. Stevens, Wood, Buchanan, and Harrison; and to the Secretaries, Drs. Arnold, Stillé, and Stewart, for the very efficient manner in which they have discharged the various duties imposed upon them.

Dr. A. Flint, of New York, presented the following Resolution, which was passed:—

*Resolved,* That it be recommended by this Convention to the medical profession of the States, wherein laws sanctioning and providing for the prosecution of dissections do not already exist, to unite in endeavouring to procure the passage of such laws.

Dr. Thomson, of Delaware, offered a Resolution relative to the organization of the "American Medical Association," but accepted as a substitute the following, presented by Dr. F. C. Stewart, of New York.

*Resolved,* That all unfinished business be referred to the "American Medical Association" about to be organized.

*Resolved,* That this Convention do now resolve itself into the "American Medical Association," and that the officers of the Convention continue to act as officers of the Association until others be appointed; which was unanimously adopted.

On motion, the President was requested to appoint a Committee, consisting of one person from each State represented in the Convention, to nominate officers of the Association for the ensuing year.

The following gentlemen were accordingly appointed:

| | | | | |
|---|---|---|---|---|
| Drs. | H. F. Askew, | *Del.* | Drs. Corbin, | *Va.* |
| " | Twitchell, | *N. H.* | " Moultrie, | *S. C.* |
| " | Hall, | *Vt.* | " Garvin, | *Ga.* |
| " | Adams, | *Mass.* | " Keirn, | *Miss.* |
| " | Dunn, | *R. I.* | " Buchanan, | *Tenn.* |
| " | N. B. Ives, | *Conn.* | " Harrison, | *La.* |
| " | J. R. Manley, | *N. Y.* | " Litton, | *Mo.* |
| " | L. A. Smith, | *N. J.* | " Frye, | *Ill.* |
| " | R. La Roche, | *Pa.* | " Shipman, | *Ind.* |
| " | Dunbar, | *Md.* | " Judkins, | *O.* |
| " | J. Riley, | *D. C.* | " Annan, | *Ky.* |

On motion of Dr. Bishop, of Connecticut, it was

*Resolved,* That the Delegates of every Society or Association, represented in this body, be requested to send to the Secretaries the form of organization, or act of incorporation, and constitution of their Society or Association with a list of its members.

Dr. Griscom, of New York, moved that the Report on the nomenclature of diseases, be referred to the Committee on Registration, appointed at the morning session. It being objected that this committee was created by the Convention, the Association, on motion of Dr. F. C. Stewart confirmed the appointment of the Committee, and the Report was referred accordingly.

Dr. Humes, of Pennsylvania, offered a resolution, directing the Committee on Registration to report on the subject of premature interments, which was laid upon the table.

The Committee appointed to nominate Officers for the American Medical Association reported the following nominations.

*President*,—Dr. Nathaniel Chapman, of Pennsylvania.

*Vice Presidents*,—Drs. Jonathan Knight, Conn.; Alexander H. Stevens, N. Y.; James Moultrie, S. C.; A. H. Buchanan, Tenn.

*Secretaries*,—Drs. Alfred Stillé, Philadelphia; J. R. W. Dunbar, Baltimore.

*Treasurer*,—Dr. Isaac Hays, Philadelphia.

It was determined, on motion of Dr. J. L. Atlee, of Pennsylvania, that the vote of the Convention for Officers be taken *viva voce*.

Dr. A. H. Stevens, of New York, moved a reconsideration of this motion, which was carried, and on motion of Dr. Askew, of Delaware, the vote was ordered to be taken by ballot. Drs. Blatchfotd, of New York, and Haxall, of Virginia, were appointed tellers, and declared that the nominees of the Committee were unanimously elected Officers of the Association for the ensuing year.

On motion of Dr. Thomson, of Delaware, a committee of five was ordered to wait upon the President elect, and invite him to take the Chair. For this purpose the President appointed Drs. Twitchell, of N. H.; E. Ives, of Conn.; Corbin, of Va.; Jervey, of S. C.; and Annan, of Ky.

Dr. J. V. C. Smith, of Mass., moved to take up his resolution in regard to the next place of meeting of the Association, and the invitation of the Delegates from Baltimore having been called for and read, it was, on motion, resolved, that their invitation be accepted.

Dr. Watson, of New York, moved for a committee of seven, to nominate members of the Standing Committees named in the plan of organization, but the motion was so amended as to empower the President and Vice Presidents, to appoint these Committees in time for the publication of the proceedings, and the motion, thus amended, was passed.

On motion of Dr. N. S. Davis, of N. Y., his resolution relative to a committee, to report on the indigenous Botany of the several states was taken from the table and adopted.

Dr. Chapman, the President elect, was then introduced, and on taking the chair, made the following remarks:

He said he could find no language to express the depth of his gratitude. It had often been his good fortune during his professional life to have been complimented in the same manner, though not in the same degree. This was the most precious of all the honours he had received, as spontaneously conferred by his own brethren. He confessed his incompetency to serve the Association as he could desire. He said he loved his profession, and should be ungrateful if he did not: whatever he possessed in this life, had been bestowed by its favours; when he forgot it, or deserted it and its disciples, he remarked with great emphasis, may Almighty God forget and desert me. He desired that the Association should be persuaded of his ardent wishes for the cause, and that his most strenuous efforts would be unceasingly directed to advance the dignity of the profession, and extend its usefulness.

The following was offered by Dr. John B. Johnson, of Missouri:

Whereas, numberless and important evils result from the almost universal practice of allowing persons wholly ignorant of drugs and medicines to engage as apothecaries; and still greater from the universal traffic in patent and secret medicines,

Therefore, resolved, that the Committee on Education inquire into the expediency of establishing a school or schools of Pharmacy in the respective States, for the special purpose of preparing persons for the business of apothecaries; and also the expediency of adopting a rule that no physician ought to patronize a druggist or apothecary who deals in patent and secret medicines—and report at the next annual meeting of the Association. Adopted.

The following was offered by Dr. F. Campbell Stewart, of New York:

*Resolved,* That the Committee on Publication be directed to have published not less than two thousand copies of the proceedings of the late Convention, with the Reports, and of this Association up to the period of its adjournment; and distribute the same to the members of the Association. Passed.

After which, on motion, the Association adjourned to meet in Baltimore on the *first Tuesday* of May, 1848.

At a meeting of the President and Vice Presidents of the "American Medical Association," held on May 8th, 1847, the following Standing Committees were appointed in pursuance of the order of the Association:

*Committee on Arrangements.*

Dr. G. C. M. Roberts, Balt., Chairman.

| | |
|---|---|
| Dr. A. C. Robinson, Balt. | Dr. Wm. Power, Balt. |
| " J. H. Briscoe, " | " W. T. Leonard, " |
| " J. R. W. Dunbar, " | " C. Bell Gibson, " |

*Committee on Medical Sciences.*

Dr. S. Henry Dickson, S. C., Chairman.

| | |
|---|---|
| Dr. J. P. Jervey, S. C. | Dr. Wm. T. Wragg, S. C. |
| " Robert Bridges, Philada. | " Wm. Power, Balt. |
| " J. W. Francis, N. Y. | " T. Romeyn Beck, N. Y. |

*Committee on Practical Medicine.*

Dr. Joseph M. Smith, N. Y., Chairman.

| | |
|---|---|
| Dr. René La Roche, Phila. | Dr. J. B. Beck, N. Y. |
| " John Harrison, La. | " Isaac Wood, " |
| " H. M. Bullitt, Mo. | " G. S. Camman, " |

*Committee on Surgery.*

Dr. George W. Norris, Philada., Chairman.

| | |
|---|---|
| Dr. Isaac Parrish, Philada. | Dr. Jacob Randolph, Phila. |
| " John Watson, N. Y. | " H. H. M'Guire, Petersburg, Va. |
| " A. L. Peirson, Salem, Mass. | " C. Bell Gibson, Balt. |

*Committee on Obstetrics.*

Dr. Harvey Lindsley, D. C., Chairman.

| | |
|---|---|
| Dr. G. C. M. Roberts, Balt. | Dr. W. Channing, Boston. |
| " J. Riley, D. C. | " C. R. Gilman, N. Y. |
| " R. W. Haxall, Richmond, Va. | " S. Annan, Lexington, Ky. |

*Committee on Medical Literature.*

Dr. Oliver Wendell Holmes, Chairman.

| | |
|---|---|
| Dr. E. Hale, Boston. | Dr. John Bell, Philada. |
| " G. C. Shattuck, Jr., " | " Austin Flint, Buffalo. |
| " D. Drake, Lexington, Ky. | " W. Selden, Norfolk, Va. |

*Committee on Medical Education.*

Dr. Alex. H. Stevens, Chairman.

| | |
|---|---|
| Dr. Amos Twitchell, Keene, N. H. | Dr. R. D. Arnold, Savannah. |
| " B. R. Wellford, Fredericksburg, Va. | " F. Campbell Stewart, N. Y. |
| | " L. P. Bush, Wilmington, Del. |
| " Arnold Naudain, Phila. | |

*Committee on Publication.*

Dr. Isaac Hays, Philada. Chairman.

| | |
|---|---|
| Dr. Alfred Stillé, Phila. | Dr. J. R. W. Dunbar, Balt. |
| " J. V C. Smith, Boston. | " Gouverneur Emerson, Phila |
| " J. P. Garvin, Augusta, Ga. | " Caspar Morris, " |

*Committee on Indigenous Botany, under the Resolution of Dr. N. S. Davis.*

Dr. N. S. Davis, Binghamton, N. Y., Chairman.

| | |
|---|---|
| Dr. S. W. Williams, Mass. | Dr. A. C. Robinson, Md. |
| " Eli Ives, Conn. | " Frederick Marx, Va. |
| " Engleman, Mo. | " J. P. Porcher, S. C. |
| " W. A. Cheetham, Tenn. | " J. Le Conte, Ga. |
| " Jos. Carson, Pa. | " Cartwright, Miss. |
| " Charles Short, Ky. | " Carpenter, La. |
| " E. E. Phelps, Vt. | " Ohio. |
| " A. Twitchell, N. H. | " G. Norwood, Ind. |
| " T. C. Dunn, R. I. | " Ill. |
| " Lyndon H. Smith, N. J. | " Mich. |
| " James Couper, Del. | " D. C. |

